# Microplastics Biodegradation by Estuarine and Landfill Microbiomes

**DOI:** 10.1007/s00248-024-02399-8

**Published:** 2024-06-28

**Authors:** Cristina S. Pires, Luís Costa, Sónia G. Barbosa, João Carlos Sequeira, Diogo Cachetas, José P. Freitas, Gilberto Martins, Ana Vera Machado, Ana J. Cavaleiro, Andreia F. Salvador

**Affiliations:** 1 CEB - Centre of Biological Engineering, University of Minho, Braga, Portugal; 2 LABBELS – Associate Laboratory, Braga/Guimarães, Portugal; 3 IPC - Institute for Polymers and Composites, University of Minho, Guimarães, Portugal

**Keywords:** PCL, PET, PE, Landfill leachate, Estuarine sediment, Biodegradation

## Abstract

Plastic pollution poses a worldwide environmental challenge, affecting wildlife and human health. Assessing the biodegradation capabilities of natural microbiomes in environments contaminated with microplastics is crucial for mitigating the effects of plastic pollution. In this work, we evaluated the potential of landfill leachate (LL) and estuarine sediments (ES) to biodegrade polyethylene (PE), polyethylene terephthalate (PET), and polycaprolactone (PCL), under aerobic, anaerobic, thermophilic, and mesophilic conditions. PCL underwent extensive aerobic biodegradation with LL (99 ± 7%) and ES (78 ± 3%) within 50–60 days. Under anaerobic conditions, LL degraded 87 ± 19% of PCL in 60 days, whereas ES showed minimal biodegradation (3 ± 0.3%). PE and PET showed no notable degradation. Metataxonomics results (16S rRNA sequencing) revealed the presence of highly abundant thermophilic microorganisms assigned to *Coprothermobacter* sp. (6.8% and 28% relative abundance in anaerobic and aerobic incubations, respectively). *Coprothermobacter* spp. contain genes encoding two enzymes, an esterase and a thermostable monoacylglycerol lipase, that can potentially catalyze PCL hydrolysis. These results suggest that *Coprothermobacter* sp. may be pivotal in landfill leachate microbiomes for thermophilic PCL biodegradation across varying conditions. The anaerobic microbial community was dominated by hydrogenotrophic methanogens assigned to *Methanothermobacter* sp. (21%), pointing at possible syntrophic interactions with *Coprothermobacter* sp. (a H_2_-producer) during PCL biodegradation. In the aerobic experiments, fungi dominated the eukaryotic microbial community (e.g., *Exophiala* (41%), *Penicillium* (17%), and *Mucor* (18%)), suggesting that aerobic PCL biodegradation by LL involves collaboration between fungi and bacteria. Our findings bring insights on the microbial communities and microbial interactions mediating plastic biodegradation, offering valuable perspectives for plastic pollution mitigation.

## Introduction

Plastics have become indispensable in modern society, playing a crucial role in various economic sectors such as agriculture and industry [1, 2]. However, their exponential production, insufficient recycling, and plastics’ slow degradation under natural conditions have resulted in the accumulation of plastic waste in ecosystems [3, 4]. Plastic waste represents 80–85% of the total marine litter [5], and a significant portion of plastic waste collected in the European Union is incinerated or buried in landfills [6]. New eco-friendly solutions for plastic waste treatment are necessary, and improved biodegradation strategies can have an important role in reducing plastic pollution.

Microplastics represent a significant environmental and health threat due to their small size and pervasive nature. Often invisible to the naked eye, microplastics are harder to detect and manage compared to larger plastic debris. They can spread more easily through the environment, reaching even the most remote areas. Moreover, microplastics can infiltrate various ecosystems, including oceans, rivers, soil, and the atmosphere, leading to widespread environmental and biological contamination. They can accumulate in the tissues of organisms, moving up the food chain and potentially impacting a wide range of species, including humans [7, 8].

Conventional plastics are synthetic and semi-synthetic polymers primarily derived from fossil carbon sources [9]. These materials are generally classified as non-biodegradable [10] and are extremely bio-inert, with a highly hydrophobic chain, which makes their biodegradation extremely difficult [1, 11, 12]. Amongst synthetic fossil-based plastics, polyethylene (PE) is the most widely produced [13], finding extensive use in the manufacture of bags, bottles, food packaging, and other products [11]. Due to the saturation of its chain with ethylene bonds, PE is a very hydrophobic polymer, and one of the most recalcitrant [13]. Although PE is considered non-biodegradable, evidence of its biodegradation was shown by complex microbial communities (e.g., waxworm gut microbiome) and pure cultures of bacteria and fungi [14–16]. However, most often, PE biodegradation occurs only partially and at very slow rate.

Polyethylene terephthalate (PET) is also a versatile polymer widely used in various everyday products [11]. The prevalence of PET in single-use plastic products makes it a significant contributor to plastic pollution [17]. Some microorganisms and enzymes were reported to biodegrade PET, but the crystalline regions are extremely resistant to biological attack [18, 19].

Due to the challenges in achieving an efficient biodegradation of recalcitrant plastics, it is worthy to replace PE and PET by biodegradable alternatives. Biodegradable plastics are polymers that undergo more easily biodegradation by microorganisms and are generally broken down into smaller molecules, such as carbon dioxide and water, with minimal production of toxic compounds [5, 20]. Poly(ε-caprolactone) (PCL) is a synthetic, fossil-based polyester and widely used biodegradable plastic [21]. PCL presents numerous benefits compared to other biodegradable plastics, leading to a rise in its applications. For example, it is resistant to water, oil, solvents, and chlorine [22]. Due to its high biocompatibility, blend compatibility, and low biodegradability rates, it is frequently used in long-term biomedical and tissue applications [22–24]. Among biodegradable plastics, PCL can have extended biodegradation times, leading to its accumulation in the environment where it is discarded [22, 25]. Total PCL biodegradation may take few months to several years, depending on the environmental conditions, as well as with variations in PCL properties (e.g., molecular weight and crystallinity) [24, 26]. Nevertheless, biodegradation has been reported to occur both in natural environments and in engineering environments (e.g., sewage sludge and compost) [24, 27], either under anaerobic or aerobic conditions [1, 28]. Recently, a PCL-degrading bacterium was isolated from a plastic-contaminated landfill and presented high biodegradation rates, showing that landfill microbiomes can efficiently biodegrade PCL [24–26].

Environmental microbiomes have the ability to adapt to external stimuli, such as plastic pollution, and may harbor microbes with the ability to biodegrade plastics more efficiently than the ones currently known. Therefore, in this work, we tested two different environmental microbiomes (landfill leachate and estuarine sediments), for their ability to biodegrade PCL, PE, and PET microplastics. These microbiomes were chosen because they originate from environments typically contaminated with plastic. In fact, despite significant efforts to recycle plastic, a substantial proportion of these materials still ends up in municipal landfills. Additionally, plastics and microplastics are abundant in marine and estuarine environments, where they often accumulate in sediments. Although PE and PET are considered recalcitrant, their microbial biodegradation has been reported, and thus, given the unexplored potential of the inocula used in our study, it was important to test their biodegradation capabilities. This work is aimed at screening microplastic biodegradation (under different incubation conditions, including aerobic, anaerobic, mesophilic, and thermophilic conditions) and identifying potential microplastic-degrading microorganisms. The temperature conditions were selected considering the origin of each inoculum, i.e., mesophilic environment for the estuarine sediment and thermophilic environment for the landfill leachate. These environments are poorly explored regarding microplastic biodegradation, and thus, this work is aimed at broadening current knowledge on the microbiology of microplastic biodegradation, opening new perspectives for the development of efficient biotechnological solutions for plastic waste treatment.

## Materials and Methods

### Plastic Materials

Pellets of PE, PET, and PCL were synthesized in the Institute for Polymers and Composites, University of Minho. Pellets were mechanically grinded to obtain particles with a 1 mm diameter.

### Inocula

Biodegradation assays were conducted using inocula leachate from the municipal landfill (Resulima) in Viana do Castelo, Portugal, and estuarine sediments collected in the Cávado river estuary near Esposende, Portugal.

The leachate was transported to the laboratory and stored at 4 °C until further analysis. Before setting up the biodegradation assays, the leachate was concentrated by decantation and centrifugation (8000 *g*, 10 min). Volatile solids (VS) were determined as described elsewhere [30]. The leachate was incubated overnight at 55 °C to consume the residual substrate.

Estuarine sediments were sampled using a 6-mm diameter PVC tube, at the layer between 2 and 12 cm depth. Sediment was transported to the laboratory at 4 °C, homogenized, and sieved (5 mm). The salinity of the seawater was 20 g L^−1^. Before starting the assays, the sediment was incubated at 37 °C under saline conditions (20 g L^−1^ of NaCl in the aerobic assay; 10 g L^−1^ in the anaerobic assays), for 2 days, for consumption of residual substrate.

### Biodegradation Assays

For both inocula, microbial degradation of plastics was investigated under anaerobic and aerobic conditions. Incubations were performed at 55–37 °C, in the assays inoculated with the leachate or the sediment, respectively. The good activity of the inocula was confirmed in control assays with microcrystalline cellulose (62.5 mg, average particle size 50 μm, Sigma-Aldrich, St. Louis, MO, USA). Methane production (in the anaerobic assays) and oxygen consumption (in the aerobic assays) started immediately, and cellulose biodegradation reached 95 ± 17% and 71 ± 11%, respectively. For the anaerobic assays, this is in agreement with the validation criterium defined by Fruteau De Laclos et al. and Holliger et al [29, 30], which states that microcrystalline cellulose conversion to methane should be 82–95% of the theoretical value.

### Anaerobic Biodegradation Assays

Anaerobic biodegradation assays were performed in 120 mL bottles containing 45 mL of bicarbonate-buffered basal medium (BM), supplemented with salts and vitamins, as described by Stams et al. [31]. The assays with the sediment were also supplemented with NaCl (10 g L^−1^). This value is lower than the salinity measured in the seawater but was chosen considering that methanogenic microorganisms generally do not tolerate high salt concentrations in the medium [32, 33].

The bottles were loaded with 62.5 mg of each plastic in powder (PE, PET, and PCL) and inoculated with 1.5 mL of leachate (corresponding to a VS concentration of 1.4 g L^−1^) or 4.5 g of sediment (VS concentration of 2.4 g L^−1^). Bottles were closed with butyl rubber septa and aluminum crimp caps, and the headspace was flushed and pressurized with N_2_/CO_2_ (80:20%, v/v, 1.7 × 10^5^ Pa). A reducing agent, Na_2_S•9H_2_O (1 mmol L^−1^), was added. These assays had no electron acceptors other than bicarbonate (methanogenic conditions). For the sediment, assays under sulfate-reducing (SR) conditions were also prepared, by adding sodium sulfate (20 mmol L^−1^). Blank tests, without the addition of plastics or any other carbon source, were also performed. All assays were conducted in triplicate, in the dark, with agitation at 150 rpm.

Methane was measured periodically. In the SR assays, sulfide was also measured to indirectly assess sulfate reduction. At the end of the incubation period, samples were collected from the assays with leachate and PCL, for VFA and microbial community composition analysis.

### Aerobic Biodegradation Assays

The aerobic experiments were performed in closed bottles, to allow for the monitoring of oxygen consumption. Basal medium was used, composed of the following stock solutions (composition of the solutions in g L^−1^): 40 mL L^−1^ of solution (A) containing KH_2_PO_4_, 28.25 and K_2_HPO_4_, 146.08; 30 mL L^−1^ of solution (B) containing CaCl_2_•2H_2_O, 3.36 and NH_4_Cl, 28.64; and 30 mL L^−1^ of solution (C) containing MgSO_4_•7H_2_O, 3.06; FeSO_4_, 0.7; and ZnSO_4_, 0.4 [34]. NaCl (20 g L^−1^) was also added to the medium in the assays with the sediment. A volume of 50 mL of medium was added to each 120 mL bottle, along with the plastics and inocula (as described for the anaerobic biodegradation assays). Blank assays (without plastics or other added carbon source) were also performed. The bottles were sealed with butyl rubber septa and aluminum crimp caps and pressurized with atmospheric air. Oxygen content in the headspace of the bottles was immediately measured. All assays were conducted in triplicate, in the dark, with agitation (150 rpm). Oxygen measurements were performed twice a week. When the oxygen levels became low, the headspace was flushed with air, and fresh air was injected using a syringe. At the end of the incubation period, samples were collected from the assays with leachate and PCL, for analysis of the microbial community composition.

### Analytical Methods

Methane (in the anaerobic assays) or oxygen (in the aerobic assays) was determined by gas chromatography (GC) using a GC BRUKER SCION 456 (Billerica, MA, USA), with a Molsieve packed column (13 × 80/100, 2 m length, 2.1 mm internal diameter) connected to a thermal conductivity detector. Argon was the carrier gas at a flow rate of 30 mL min^−1^, with temperatures of 100 °C, 35 °C, and 130 °C for injector, column, and detector, respectively.

For total dissolved sulfide analysis, samples were collected, immediately transferred to a zinc acetate solution (2% w/v), and analyzed with Hach cuvette tests (LCK653) and a DR 2800 spectrophotometer (Hach, Düsseldorf, Germany [35].

VFA analyses were carried out using HPLC equipment (Jasco, Tokyo, Japan) with a Rezex ROA-Organic Acid H^+^ (8%) LC Column (300 × 7.8 mm) at 60 °C. The mobile phase comprised a solution of sulfuric acid (5 mmol L^−1^), and crotonic acid was utilized as the internal standard. Elution was performed at a flow rate of 0.6 mL min^−1^, and compound detection occurred at 210 nm [36].

### Data Analysis

Under anaerobic conditions, the biodegradability of the microplastics was determined considering the experimentally measured values of CH_4_ (BMP_exp_) and the theoretical biochemical CH_4_ production (BMP_theoric_), according to Eq. 1.

1 $$\mathrm{B}\mathrm{i}\mathrm{o}\mathrm{d}\mathrm{e}\mathrm{g}\mathrm{r}\mathrm{a}\mathrm{d}\mathrm{a}\mathrm{t}\mathrm{i}\mathrm{o}\mathrm{n} \left({\%}\right)=\frac{{\mathrm{B}\mathrm{M}\mathrm{P}}_{\mathrm{e}\mathrm{x}\mathrm{p}.}}{{\mathrm{B}\mathrm{M}\mathrm{P}}_{\mathrm{t}\mathrm{h}\mathrm{e}\mathrm{o}\mathrm{r}\mathrm{i}\mathrm{c}}} \times 100$$

The BMP_theoric_ was obtained from the element composition of the microplastics (C, H, N, O, and S), according to Reaction 1 and Eq. 2 [37]. BMP_theoric_ was expressed in volume of methane (L) at standard temperature and pressure (STP) per mass unit of plastic (g). The BMP_theoric_ values calculated for the different plastics in the study are shown in Table 1.

**Table 1:** Theoretical biochemical CH_4_ potential (BMP_theoric_) for the different plastics in the study

Plastic	Formula	BMP_theoric_
		L g^−1^
PE	C_2_H_4_	1.20
PET	C_10_H_8_O_4_	0.58
PCL	C_6_H_10_O_2_	0.74

**Reaction 1** 

$${\mathrm C}_{\mathrm x}{\mathrm H}_{\mathrm y}{\mathrm O}_{\mathrm z}{\mathrm N}_{\mathrm d}{\mathrm S}_{\mathrm e}+\left(x-\frac y4-\frac z2+\frac{3d}4+\frac e2\right){\mathrm H}_2\mathrm O\rightarrow\left(\frac x2+\frac y8-\frac z4-\frac{3d}8-\frac e4\right){\mathrm{CH}}_4+\left(\frac x2-\frac y8+\frac z4+\frac{3d}8+\frac e4\right){\mathrm{CO}}_2+\mathrm d{\mathrm{NH}}_3+{\mathrm{eH}}_2\mathrm S$$

2 $${\mathrm{B}\mathrm{M}\mathrm{P}}_{\mathrm{t}\mathrm{h}\mathrm{e}\mathrm{o}\mathrm{r}\mathrm{i}\mathrm{c}}=\frac{22.4\left(\frac{\textit{x}}2+\frac{\textit{y}}8-\frac{\textit{z}}4-\frac{3\textit{d}}8-\frac{\textit{e}}4\right)}{12\textit{x}+\textit{y}+12\textit{z}+14\textit{d}+32\textit{e}}$$

Under aerobic conditions, the microplastics biodegradation (%) was calculated according to Eq. 3, where the O_2,sample_ and O_2,blank_ are the experimentally measured values of O_2_ consumed in sample and blank assays, respectively.

3 $$\mathrm{B}\mathrm{i}\mathrm{o}\mathrm{d}\mathrm{e}\mathrm{g}\mathrm{r}\mathrm{a}\mathrm{d}\mathrm{a}\mathrm{t}\mathrm{i}\mathrm{o}\mathrm{n} \left({\%}\right)=\frac{({\mathrm{O}}_{2,\mathrm{s}\mathrm{a}\mathrm{m}\mathrm{p}\mathrm{l}\mathrm{e}}-{\mathrm{O}}_{2,\mathrm{b}\mathrm{l}\mathrm{a}\mathrm{n}\mathrm{k}})}{\mathrm{T}\mathrm{h}\mathrm{O}\mathrm{D}} \times 100$$

The theoretical O_2_ demand (ThOD) for complete aerobic mineralization of the polymer C_c_H_h_O_o_ (expressed as mass of O_2_ per mass of polymer) was calculated according to Eq. 4, where *M*_*r*_ corresponds to the relative molecular mass of the polymer. The ThOD values determined for the different plastics in the study are shown in Table 2.

4 $$\mathrm{T}\mathrm{h}\mathrm{O}\mathrm{D}=\left(\frac{31.9988}{{\textit{M}}_\textit{r}}\right)(\textit{c}+0.25\textit{h}-0.5\textit{o})$$

**Table 2:** Theoretical oxygen demand (ThOD) for the different plastics in the study

Plastic	Formula	ThOD
		g g^−1^
PE	C_2_H_4_	3.43
PET	C_10_H_8_O_4_	1.67
PCL	C_6_H_10_O_2_	2.11

### Microbial Community Composition

Microbial community composition was studied at the end of the PCL biodegradability assays inoculated with the landfill leachate, as well as in the inoculum leachate. Both prokaryotic and eukaryotic communities were investigated. Total DNA was isolated, and 16S rRNA and 18S rRNA genes were sequenced by Illumina MiSeq [38]. The primers used were the following: 515F (5′-GTGCCAGCMGCCGCGGTAA-3′) and (806R: 5′-GGACTACHVGGGTWTCTAAT-3′) [39], targeting the prokaryotic community, and EUK1391F (5′-GTACACACCGCCCGTC-3′) and EUKBR (5′-TGATCCTTCTGCAGGTTCACCTAC3′) [40] targeting the eukaryotic community. Sequencing and bioinformatics analyses were performed by RTL Genomics (Lubbock, TX, USA), and the detailed methodology is described by Salvador et al. (2019) [38]. The database used for taxonomic assignment consisted of high-quality sequences derived from NCBI that are maintained in the Research and Testing Laboratory (http://www.medicalbiofilm.org).

The FASTQ files were submitted to the European Nucleotide Archive (ENA) under the accession number PRJEB73311.

### Mining *Coprothermobacter *Species Genomes for PCL Degrading Enzymes

The M-party tool (https://anaconda.org/bioconda/m-party) was harnessed to conduct an in-depth exploration of proteins potentially homologous to carboxylesterases, lipases, and cutinases, previously reported as capable to hydrolyze PCL [41–43]. The search was performed in the genomes of *Coprothermobacter* species, the ones which have their genomes sequenced (taxonomy_id:55,509, comprising the genome of *Coprothermobacter proteolyticus* (strain ATCC 35245and *Coprothermobacter* sp.), which are the closer relatives of the *Coprothermobacter* microorganisms found in our leachate sample. M-party compiled multiple enzymes obtained from the KEGG database corresponding to the EC numbers 3.1.1.1 (4466 enzymes), 3.1.1.3 (11,425 enzymes), and 3.1.1.74 (1350 enzymes). Clustering of sequences was performed with CD-HIT [44], using default parameters and an identity threshold of 70%. Multiple sequence alignment for each cluster was done with T-Coffee [45]. Finally, hidden Markov models (HMMs) were constructed with the multiple sequencing alignments by utilizing HMMER3 [46] using default settings.

## Results and Discussion

### Anaerobic Biodegradation Assays

**Fig. 1:**
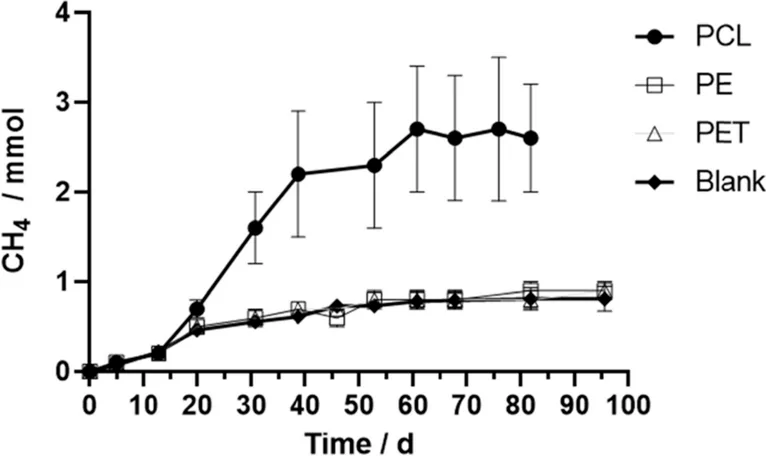
Cumulative methane production in the assays containing different microplastics, inoculated with landfill leachate and incubated at 55 °C. Cumulative methane production in the blanks is also shown. Each data point represents the average of triplicates ± standard deviation

Under anaerobic conditions, landfill leachate extensively biodegraded PCL with concomitant methane production. Cumulative methane production is represented in Fig. 1. After 60 days of incubation, the methane produced accounted for 87 ± 19% of the value that could be expected from complete PCL degradation (Table 1). These results are in accordance with the literature, with other authors reporting 80–92% PCL biodegradation in similar time periods [47–49]. A lag phase of approximately 10 days preceded the onset of methane production from PCL, probably due to microbial adaptation to the polymer and/or incubation conditions. The methane produced during the first 10 days of incubation in these assays closely followed the results of the blanks, pointing out that it was derived from the consumption of residual substrate still present in the inoculum. No VFA was detected at the end of the experiments.

For the estuarine sediment, PCL degradation started after 20 days of incubation (data not shown) and after 60 days corresponded only to 3.3 ± 0.3% biodegradation (considering the BMP_theoric_ in Table 1) (data not shown). This residual methane production can be attributed to the low methanogenic activity of the sediment. Indeed, estuarine environments are intermittently exposed to air and present relatively high salinity which does not favor the growth of strict anaerobes like methanogens [32, 33]. Low PCL biodegradation (2%) was also previously reported in batch assays, in this case using anaerobic sludge as inoculum, after 56 days of incubation [50]. In the assays with the sediment and sulfate, no sulfide production was observed throughout the experiment, pointing to the absence of sulfate-reducing microorganisms capable of degrading PCL.

Contrary to what was observed with PCL, in the assays with PE or PET cumulative methane production closely followed that of the blanks over all the experiment (Fig. 1 for leachate; data not shown for sediment). These results suggest that the methane produced was most likely derived from the consumption of residual substrates rather than from the biodegradation of the polymers. Similar results were obtained by other authors [51, 52]—for example, Selke et al. [51] reported that after 500 days of incubation, biogas production in assays containing these polymers did not exhibit a significant difference compared to the blank. When the sediment was incubated under sulfate-reducing conditions, no polymer biodegradation coupled with sulfate reduction was observed as well.

The disparity observed between PCL and the other tested polymers can be attributed to its higher susceptibility to microbial hydrolysis. Additionally, it presents a lower melting point (around 60 °C) [28], while PET is more susceptible to microbial attack within the temperature range of 75 to 80 °C [53]. These physical-chemical properties are a distinctive factor, resulting in different degradation profiles [24, 26].

### Aerobic Biodegradation Assays

**Fig. 2:**
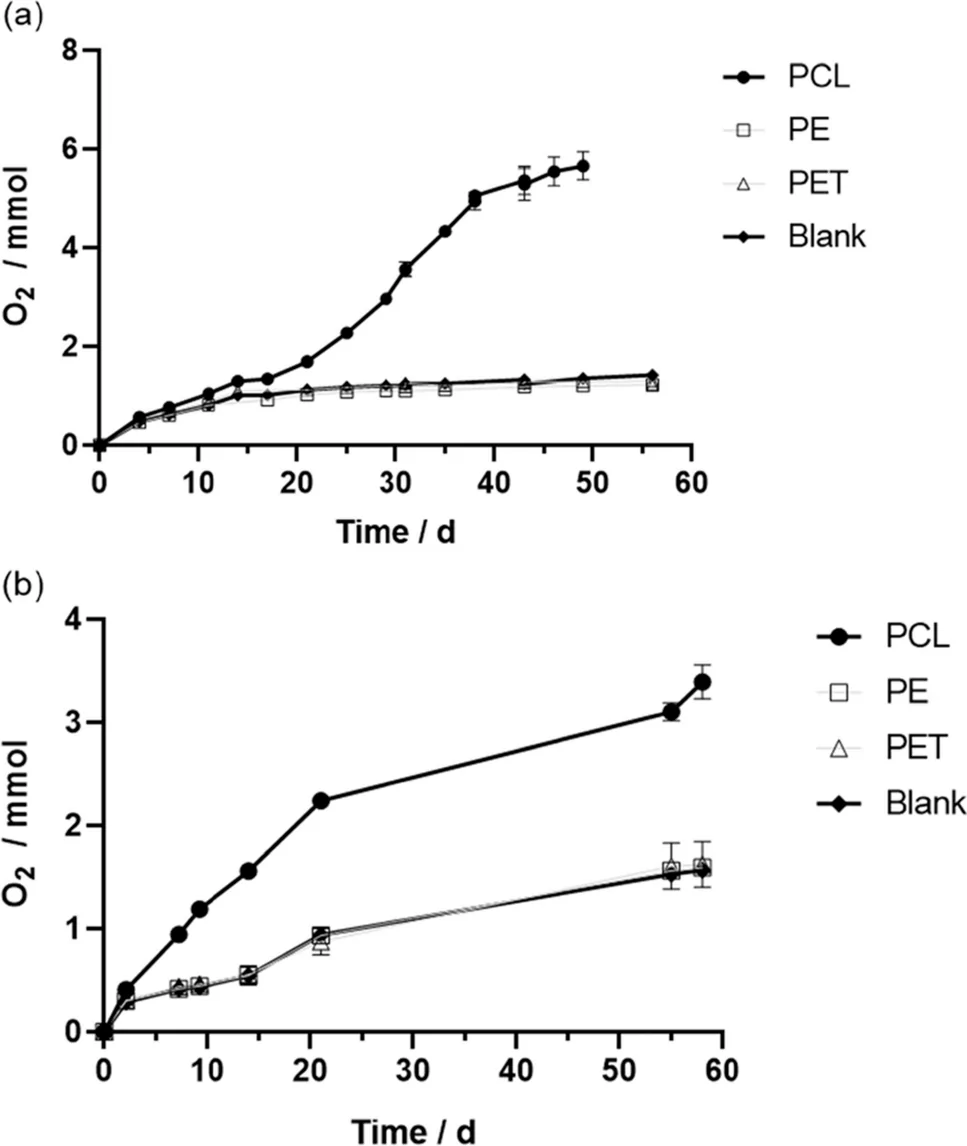
Cumulative O_2_ consumption in the assays containing different microplastics, inoculated with **a** landfill leachate (55 °C) and **b** estuarine sediment (37 °C). Cumulative O_2_ consumption in the blanks is also shown. Each data point represents the average of triplicates ± standard deviation

In the aerobic incubations, similarly to the anaerobic assays, PCL was the only microplastic biodegraded. PCL was consumed by the two inocula, as shown by the cumulative oxygen consumption curves obtained for the landfill leachate (Fig. 2a) and the estuarine sediment (Fig. 2b). Considering the ThOD values (Table 2), 99 ± 7% (50 days) and 78 ± 3% (63 days) of PCL mineralization were achieved by the leachate and the sediment, respectively.

Because PCL is a biodegradable plastic, it is expected that it should undergo biodegradation by environmental microbiomes and also under composting conditions. However, the efficiency of PCL biodegradation varies depending on the conditions applied and probably on the microbial composition of the microbiomes. Results similar to the ones obtained in our study were reported by other authors [24, 54], but, on the other hand, under aerobic composting conditions, Pradhan et al. [55] achieved only 60% biodegradation after 180 days. Variability in the results has also been reported across different studies regarding PCL biodegradation in marine environments, which were attributed to variations in experimental conditions, such as the use of artificial seawater and different sediment types (coastal or fluvial) [56].

Regarding PE and PET assays, both for inocula, the results obtained were similar to those observed in the blanks assays, revealing the lack of polymers’ biodegradation under aerobic conditions. This is in line with literature results, where mineralization was not observed with powdered PE during simulated composting at 58 °C under aerobic conditions [51].

### PCL-Degrading Thermophilic Microbiomes

More extensive biodegradation of PCL was achieved in the assays inoculated with landfill leachate than in the ones with estuarine sediment, for a similar period. This fact might be related to the origin of each inoculum, and therefore, the composition of the microbial communities, as well as with the different experimental conditions applied (e.g., mesophile or thermophile temperature). Estuarine sediment is a natural inoculum, eventually contaminated by microplastics [57]. Landfill leachate results from a myriad of physical-chemical and biological processes during the decomposition of municipal wastes and is characterized by a miscellany of organic and xenobiotic compounds that induce a microbial community with the potential to present high diversity and metabolic functions [58].

Based on the promising results obtained with the landfill leachate, and considering that it originates from an understudied environment, with significant potential for harboring novel plastic-degrading microorganisms, this microbial community was further studied. Moreover, it is a thermophilic community, and not much is known about thermophilic microorganisms that can degrade plastic, which further reinforces the interest of this study. Utilizing thermophiles in biotechnological processes offers several advantages, namely, decreased viscosity of the medium and enhanced bioavailability and solubility of organic compounds, which results in higher reaction rates [59]. The use of stable enzymes at high temperatures prolongs hydrolysis of the polymers’ backbone [59, 60]. There are some known thermophilic microorganisms able to biodegrade PCL [60, 61], and leachate may be a source of microbes yet unknown with this capability.

**Fig. 3:**
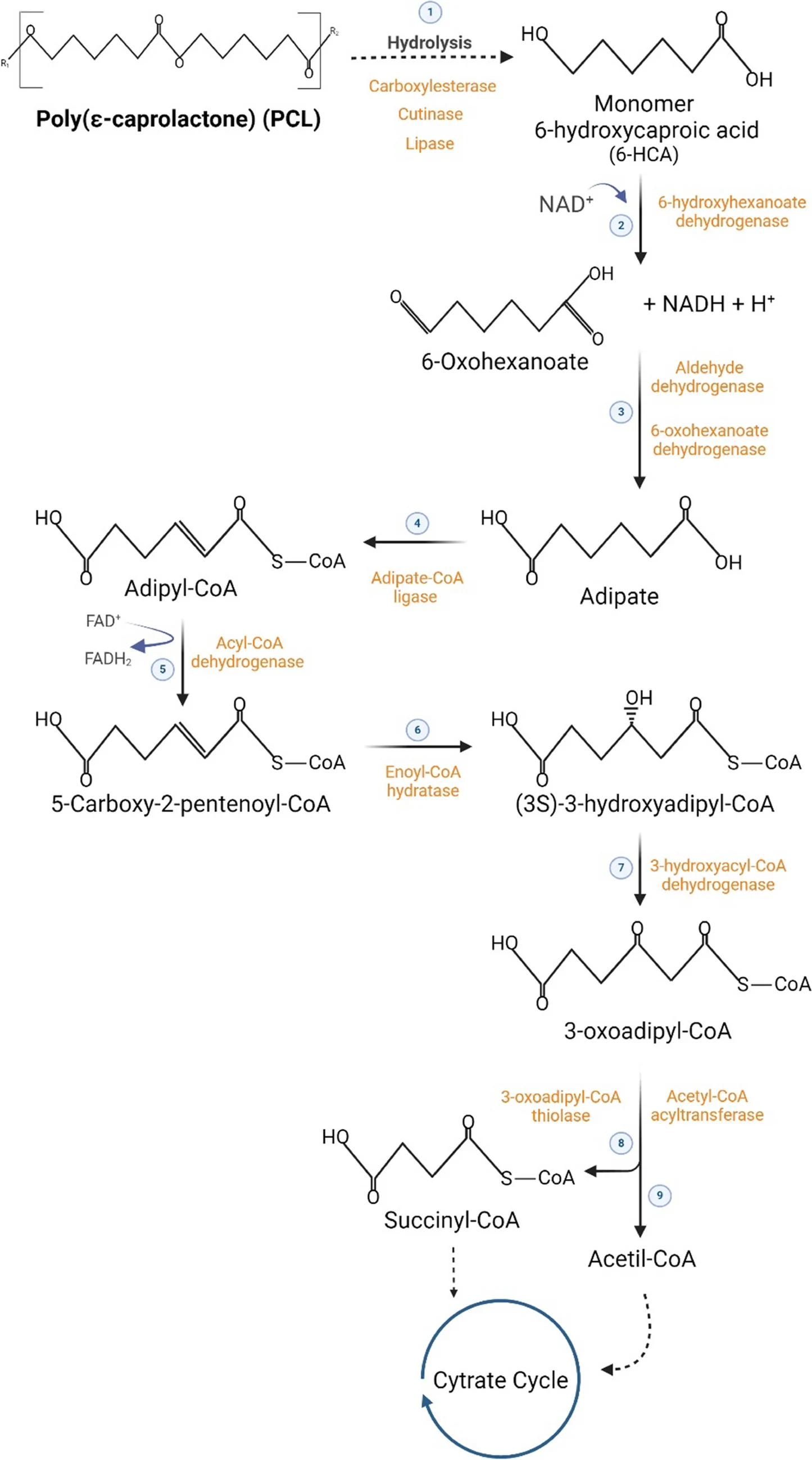
Possible pathway for PCL biodegradation, based on [62, 69] and on information from KEGG database (maps 00930 and 00362)

PCL biodegradation involves several steps, starting with the hydrolysis of the polymer, which is usually the most difficult step. After hydrolysis, PCL monomers (6-hydroxycaproic acid) may undergo a cascade of reactions which finalizes in the TCA cycle [62, 63] (Fig. 3). It is still unknown whether this pathway occurs under thermophilic conditions and if it is performed by the majority of PCL biodegraders.

### Anaerobic Community

**Fig. 4:**
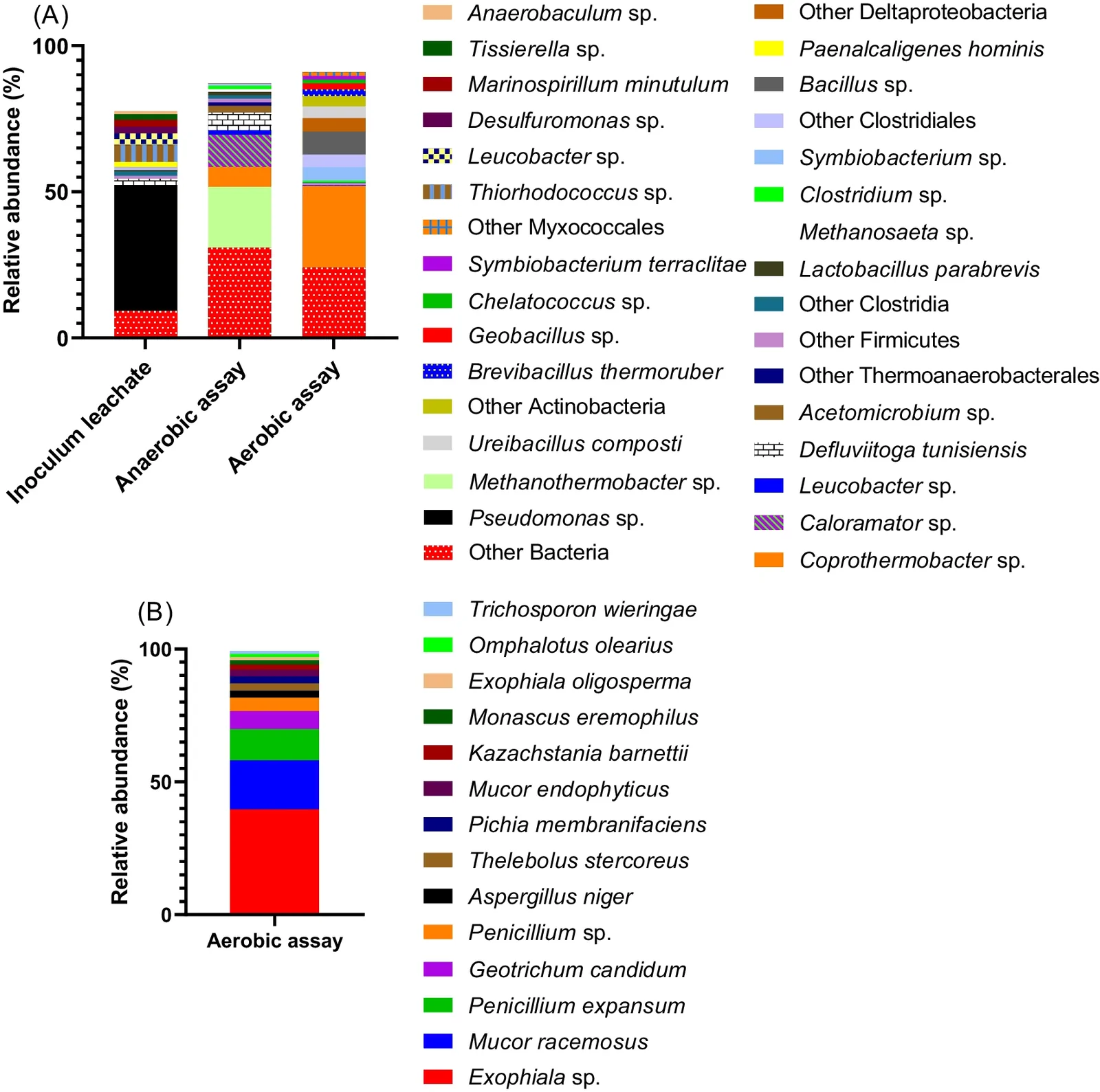
Taxonomic characterization and relative abundance of the **A** prokaryotic microorganisms, given by 16S rRNA gene sequencing, of landfill leachate before (inoculum) and after the anaerobic and aerobic PCL biodegradation assays; and of the **B** eukaryotic microorganisms assigned to the fungi kingdom, obtained by 18S rRNA amplicon sequencing, at the end of the aerobic PCL biodegradability assays. Only OTU's with relative abundance higher than 1% were considered

Figure 4**A **displays the microbial community composition and the relative abundance of the microorganisms identified in the anaerobic assays with landfill leachate and PCL. Bacteria and Archaea accounted for 77% and 23% of the classified organisms, respectively. In a significant proportion of the retrieved sequences (~31%), taxonomic identification was only possible at the kingdom level (bacteria), possibly due to the scarce knowledge of plastic-biodegrading microorganisms and leachate microbiomes. Microorganisms from the *Methanothermobacter* (20.9% relative abundance), *Caloramator* (11.0%), *Coprothermobacter* (6.8%), *Defluviitoga* (6.1%), *Acetomicrobium* (2.3%), and *Leucobacter* (1.5%) genera predominated in the community, accounting for a total of 49% of the retrieved sequences.

Microorganisms assigned to the *Methanothermobacter* genus are thermophilic and produce methane by utilizing hydrogen as an energy source [64, 65]. Its high abundance in the anaerobic assays suggests that hydrogenotrophic methanogenesis has an important role in PCL conversion to methane. Still, *Methanosaeta* sp. (1.1% relative abundance, Fig. 4A) and *Methanosarcina* (0.5%) were also present in the community, showing that both hydrogenotrophic and acetoclatic pathways were occurring during PCL conversion to methane.

*Coprothermobacter* spp. are anaerobic thermophilic bacteria that possess substantial enzymatic activity, both intracellularly and extracellularly, particularly in proteolytic capabilities [66]. Moreover, these microbes are hydrogen producers and have been reported to facilitate interspecies hydrogen transfer and accelerate protein degradation when in co-culture with *Methanothermobacter* sp [66]. Jin et al. [49] reported a positive correlation between the relative abundance of *Methanothermobacter* sp. and *Coprothermobacter* sp. during the degradation of biodegradable plastics, including PCL. Similar syntrophic interactions can possibly be occurring in the anaerobic assays performed in this work with the landfill leachate and PCL, revealing novel microbial interactions in microplastic biodegradation.

Bacteria from the genus *Caloramator* (11% relative abundance) have not been associated with PCL biodegradation before [67, 68] but are able to ferment a wide range of substrates, including several carbohydrates derived from plant biomass. Therefore, their possible role in the hydrolysis of PCL may be hypothesized.

*Lactobacillus* spp. and *Pseudomonas* spp. were also identified in the anaerobic community, with 1.3% and 0.6% relative abundance, respectively (Fig. 4A). Bacteria from these two genera have been reported to secrete extracellular enzymes, such as lipases and esterases, which can break down the chemical bonds of PCL [72–74]. Furthermore, *Pseudomonas* spp. can also degrade PCL via intracellular enzymes [53]. Although being present with low relative abundance in the anaerobic community, these bacteria may also have a role in PCL degradation. Although preferring aerobic metabolism, bacteria from the *Pseudomonas* genus are capable to grow under anaerobic conditions, using nitrate or nitrite as electron acceptor [70]. *Pseudomonas* are also able to ferment arginine and pyruvate anaerobically [71, 72]. Arginine fermentation leads to very slow growth [73], while pyruvate fermentation has been associated with long-term survival and does not seem to contribute to anaerobic cell growth [72]. Substrate level phosphorylation coupled with the reduction of electron shuttles, such as phenazines, is another strategy described to promote energy conservation pathways that facilitate *Pseudomonas*’ anaerobic survival [74, 75]. Bearing in mind this highly versatile energy metabolism of *Pseudomonas*, it seems adequate not to rule out any possible role for *Pseudomonas* in PCL biodegradation under anaerobic conditions. Still, it is important to notice that *Pseudomonas* was the predominant genus identified in the leachate (42.9% relative abundance, Fig. 4A), but when incubated with PCL under strict anaerobic conditions, its relative abundance sharply decreased.

### Aerobic Assays

The prokaryotes identified in the aerobic microbial community degrading PCL, as well as in the inoculum leachate, and their relative abundances, are displayed in Fig. 4A. A predominance of microorganisms from the bacteria domain was observed, accounting for 99.8% of the total diversity. Among the bacteria present in the samples, *Coprothermobacter* sp. was the most abundant, comprising 28% of the total identified community. Additionally, several groups belonging to the phylum *Firmicutes* constituted 62.6% of the total community, and other genera of bacteria were also present in relevant proportions, including *Bacillus* (8.9%), *Symbiobacterium* (6%), *Ureibacillus* (4.3%), *Brevibacillus* (2.3%), and *Geobacillus* (2%).

The hydrolytic function of *Coprothermobacter* on PCL has been previously hypothesized by Jin et al. [49] although conclusive evidence is still lacking. However, considering the significantly high relative abundances of *Coprothermobacter* sp. in the incubations with PCL (6.8% in anaerobic assays and 28% in aerobic assays), it strongly suggests that these microorganisms indeed play a significant role in the PCL biodegradation process. *Coprothermobacter* has been found to be one of the predominant bacteria in petroleum reservoirs [49], suggesting its capability to deal with recalcitrant compounds.

Inspection of the genomes of *Coprothermobacter* species revealed the presence of two enzymes that can potentially catalyze the hydrolysis of PCL. A total of 5374 HMMs were generated from enzyme sequences of carboxylesterases, lipases, and cutinases. The enzymes found were an esterase (Uniprot ID: A0A922ZSW3) from *Coprothermobacter* sp. and a thermostable monoacylglycerol lipase (Uniprot ID: B5Y789) *from Coprothermobacter proteolyticus* (strain ATCC 35,245), matching 52 and 71 HMMs derived from carboxylesterases (EC 3.1.1.1), respectively, with *E*-values ranging from 1.4*E−*88 to 1.1E−53.

Care should be taken with conclusions relying on the analysis of genomes of closely related species, since functional and physiological heterogeneity among species of the same genus may occur. This means that the *Coprothermobacter* present in leachate samples may have different hydrolytic enzymes in its genome or may have none. Also, despite the high homology between the hydrolytic enzymes found in the genomes of *Coprothermobacter* species to those previously associated with plastic biodegradation, enzyme activities and affinities to PCL as substrate need to be tested to unequivocal conclude about their function.

Nevertheless, these data taken together, i.e., the high abundance of *Coprothermobacter* species in aerobic and anaerobic assays where PCL was the only carbon and energy source, and the existence of hydrolytic enzymes (very close to carboxylesterases, lipases, and cutinases, previously reported as capable to hydrolyze PCL [41–43]), in the genomes of two *Coprothermobacter* species, suggests a role on PCL degradation and motivates further studies targeting the isolation of the species and testing its biodegradation activity towards PCL and other plastics.

Despite *Coprothermobacter* spp. are considered strictly anaerobic microorganisms, this bacterium was present in high abundance in the aerobic assays. Although it was never reported, *Coprothermobacter* spp. may be tolerant to oxygen, which might explain their capacity to endure in this community, where other strict anaerobic microorganisms could not (e.g., *Methanothermobacter* sp.).

Among the different bacterial genera identified in the community (Fig. 4A), the genus *Bacillus* (9% relative abundance) [76, 77] and *Geobacillus* (2%) [78] hav been previously associated with PCL biodegradation.

Besides bacteria, fungi were also present in this PCL-degrading community. Several eukaryotic microorganisms assigned to the fungi kingdom were identified by 18 S amplification (Fig. 4B).

The results indicate that the phylum Ascomycota dominates, accounting for 76.9% of the identified eukaryotic community (Fig. 4B). Within this phylum, the genera *Exophiala* (40.8%), *Penicillium* (16.9%), *Aspergillus* (2.7%), and *Monascus* (1.6%) are the most prevalent. *Exophiala* species were reported to degrade polyurethane (PU), a non-biodegradable polymer [79, 80]. On the other hand, *Penicillium* is well-known for its ability to degrade various plastics, particularly biodegradable polymers like PCL [81].

*Mucor racemosus* (18.4%), from the phylum Mucoromycota, is the second most abundant fungi identified in this community. Although no evidence was found in the literature regarding its ability to biodegrade PCL, some studies report its ability to degrade other polymers such as polyvinyl chloride (PVC) [82], polybutylene succinate (PBS) [83], and even crude petroleum by-products [84].

Several genera belonging to the order Saccharomycetales have also been identified, including *Geotrichum* (6.7%), *Pichia* (2.7%), *Thelebolus* (2.7%), and *Kasachstania* (2%). While there is limited literature available on the role of *Geotrichum* on PCL biodegradation, it has been reported to possess degrading properties towards other plastics, such as polycarbonate (PC) [84] and polyhexamethyleneguanidine (PHMB) [85].

Among the identified microorganisms, fungi are widely recognized as significant lipase-producers. Nakajima-Kambe et al. [86] reported that purified native and recombinant lipases from *Aspergillus niger* (2.7% relative abundance in the community) could degrade PCL.

All these results indicated that fungi and bacteria, especially *Coprothermobacter* sp., may be involved in PCL degradation in the aerobic assays, working individually or interacting within this complex microbial network.

## Conclusion

In conclusion, this study demonstrates the potential of a landfill leachate and estuarine sediment as sources for biodegrading PCL. PCL biodegradation under both anaerobic and aerobic thermophilic conditions within approximately 50 days, using leachate as inoculum, as well as estuarine sediment in mesophilic aerobic conditions. However, the more recalcitrant polymers, PE and PET, did not show significant biodegradation. Taxonomic analysis of samples from the aerobic and anaerobic assays with PCL revealed the prominence of *Coprothermobacter*, and we found that *Coprothermobacter* species contain genes coding for enzymes that can potentially hydrolyze PCL. Also, given the high predominance of *Methanothermobacter*, it is likely that these two species collaborate in PCL biodegradation processes under methanogenic conditions.

These findings contribute to our understanding of the biodegradation potential of PCL and shed light on the microbial communities involved in the degradation process. The insights gained from this study could inform the development of more sustainable approaches for plastic waste management and highlight the importance of considering specific environmental conditions and microbial interactions in biodegradation studies.

## Data Availability

Nucleotide sequencing data have been submitted to the European Nucleotide Archive (ENA) under the study accession number PRJEB73311.
